# Development of Leadership Skills During Anatomy Small-Group Sessions in a Pre-clerkship Medical Curriculum

**DOI:** 10.1007/s40670-025-02391-y

**Published:** 2025-04-14

**Authors:** Phoebe L. Li, Rijul Asri, George Holan, Christin Traba, Sophia Chen, Jeremy J. Grachan

**Affiliations:** 1 Office of Education, Rutgers New Jersey Medical School, B- 517 Medical Science Building, 185 South Orange Avenue, Newark, NJ 07103 USA; 2 Department of Medicine, Rutgers New Jersey Medical School, Newark, NJ USA; 3 Department of Surgery, Rutgers New Jersey Medical School, Newark, NJ USA; 4 Department of Pediatrics, Rutgers New Jersey Medical School, Newark, NJ USA; 5 Department of Psychiatry, Rutgers New Jersey Medical School, Newark, NJ USA

**Keywords:** Anatomy education, Small-group learning, Group facilitation, Leadership, Medical students

## Abstract

There is a demand for leadership development within undergraduate medical education. This study explored a student peer facilitator role within a pre-clerkship anatomy small-group curriculum to evaluate its impact on leadership skill development. Facilitators led their group during discussions, and the role rotated between group members after each quiz. Data were collected through paired pre- and post-course surveys, post-session summary forms, and individual reflection surveys. Facilitators reported significantly gaining confidence in various leadership skills (*p* = 0.007). This suggests that a peer facilitator role could provide students with valuable leadership experience, and future studies could include more structured training and explicit feedback.

**Supplementary Information:**

The online version contains supplementary material available at 10.1007/s40670-025-02391-y.

## Background

Physicians often take on leadership roles across various job types regardless of their field of specialization; thus, it is critical that doctors develop effective leadership skills during their training to ensure high-quality patient care [1]. It is also crucial that these skills are taught early in a physician’s education, with consistent opportunities to practice and improve [2]. To this end, there is an increased demand for structured, longitudinal experiences in leadership development within undergraduate medical education [2]. There have been efforts to introduce standardized curricula with early exposure to leadership through structured experiences, such as leadership-related internships [3]. However, this often necessitates large curricular reforms, such as building a separate course or greatly changing the structure of the pre-existing curriculum [4]. As such, integration into pre-existing curriculum is important, as this can reduce the resources and time required to implement such an opportunity.

One approach is the addition of a student facilitator role to pre-existing small-group learning sessions, which could create a consistent, longitudinal opportunity to practice leadership without a substantial curricular change [5]. A group facilitator may promote more structured group work through increased interaction and give less-vocal students an opportunity to develop leadership and interpersonal competencies [6, 7]. Longitudinal experiential learning is an effective way to develop leadership skills, and the addition of this role within a core curricular component rather than an elective ensures all students are able to experience this opportunity [8].

## Activity

This study aimed to explore the impact of a student facilitator role on leadership skill development, which was introduced to the Class of 2026 small-group anatomy curriculum at Rutgers New Jersey Medical School (NJMS). This anatomy component is integrated into the organ system-based courses of the pre-clerkship curriculum, which spans two academic years.

Students (*n* = 178) who completed an informed consent were followed through the anatomy component of their body systems-based pre-clerkship curriculum during their first two academic years. This course utilizes small-group sessions, with students discussing content related to gross anatomy, embryology, and histology that is covered in the instructor-created assigned readings. Students also complete clinical case activities and dissections of human anatomical donors. For every 1–3 sessions, students complete multiple-choice question summative quizzes. There are 14 total quizzes. The students first completed the quiz individually and then again as a group such that they could work together on the questions.

The 30 six-student groups remained together for the 26 sessions. For the NJMS Class of 2026, a new same-level peer group facilitator role was added to the small-group learning format, and while participation in the study was optional, this role was a required component of the curriculum. Thus, all students were instructed to rotate through this role after every quiz such that each student held this position for roughly four sessions. Students were given verbal instructions that described this role’s responsibilities, including moderating group dynamics during discussions and leading during the educational activities of their designated sessions. Students were instructed to avoid teaching the content to their group, as all group participants were still required to complete the prerequisite reading. Students were not given further instructions beyond these broad guidelines.

Data were collected through voluntary completion of a demographic survey, pre-course and post-course surveys, session summary forms, and individual reflection surveys sent after each quiz. The two surveys contained identical questions about self-perceived leadership skills to identify any changes that may have resulted from serving as a facilitator throughout the curriculum. Students were asked to rate their confidence on a 5-point scale, with 1 indicating no confidence and 5 indicating very confident. The survey items were chosen to measure learning outcomes identified by the Medical Leadership Competency Framework (MLCF), and the measured themes fall under the following MLCF competencies: Demonstrating Personal Qualities, Working with Others, and Managing Services [9].

As part of the curriculum, facilitators were asked to complete a session summary form at the end of each session (Supplemental 1), which collected information on the involvement of group members in the discussions. This form also provided the facilitators with a few suggested techniques for balancing group dynamics. Additionally, all students were asked to complete individual reflection surveys after each quiz, which asked students about their role during the sessions and any benefits or challenges associated with that role. Confidence in perceived leadership abilities, attitudes towards leadership, and student perceptions of coursework have been previously identified as common assessment tools to measure outcomes for curricular changes [8].

All surveys were reviewed by four faculty members and students. Data were deidentified and analyzed in IBM Statistical Package for the Social Sciences (SPSS) Version 29 (International Business Machines (IBM), Armonk, NY), and the α-level was set at 0.05. All free-text responses were independently reviewed to identify common qualitative themes by two co-authors. There were only minor discrepancies, which were discussed and resolved by consensus.

## Results and Discussion

### Leadership Development

This study sought to explore students’ perceived confidence with aspects of leadership and if these perceptions changed after serving as the group facilitator several times in the anatomy curriculum. Out of demographic survey respondents (*n* = 61), only 13% reported no previous tutoring or teaching experience. Only students who completed both the pre- and post-course surveys were included in this analysis (*n* = 23, 12.9%, Table 1). Students reported significantly increased confidence in helping a team stay focused (pre-course: *M* = 3.87, SD = 0.82; post-course: *M* = 4.26, SD = 0.75; Wilcoxon signed ranks test *Z* = − 2.714, *p* = 0.007). Similarly, there was a statistically significant increase in confidence with ensuring that a team works well together completing tasks (pre-course: *M* = 4.00, SD = 0.74; post-course: *M* = 4.39, SD = 0.66; *Z* = − 2.714, *p* = 0.007). Students also reported increased confidence in the other four questions, but there was no significant difference.

**Table 1:** Comparison of pre-course and post-course responses regarding leadership skills in the Class of 2026

Questions	Pre-course (*n* = 23)		Post-course (*n* = 23)		*p*-value^b^	Cohen’s *D*
	Mean	Stdev^a^	Mean	Stdev		
1: How confident are you with helping a team stay focused?	3.87	0.82	4.26	0.75	0.007*	0.50
2: How confident are you in ensuring a team works well together completing tasks/assignments?	4.00	0.74	4.39	0.66	0.007*	0.56
3: How confident are you in ensuring the team members are participating equally on group tasks/assignments?	3.78	0.85	3.91	0.90	0.491	0.15
4: How confident are you with addressing team members who are underperforming on a team (e.g., coming unprepared to class, late to sessions, not participating in discussions)?	3.26	1.18	3.70	1.06	0.092	0.39
5: How confident are you overall with leading a team?	3.96	0.71	4.22	0.74	0.058	0.36
6: How confident are you that your experience as a facilitator will help you gain skills to prepare you for working with teams in future settings?	3.57	0.99	3.87	1.22	0.191	0.27

^a^Standard deviation

^b^Wilcoxon signed ranks test

Based on the summary forms (*n* = 485), most groups reported equal participation during the discussion, with only 3 groups reporting unequal participation per session on average. The summary form also asked how facilitators handled varied participation, and the most common technique was to directly ask a quieter student to answer a question. There were 12 instances where the facilitator directly spoke with under-participating members regarding their participation, which suggests that some students felt comfortable providing direct constructive feedback to their peers. Of the free-text responses, common themes included systematically having students taking turns when answering questions and confirming understanding from all members through verbal check-ins.

### Student Perceptions

Surveys were sent throughout the entire curriculum after every facilitator rotation (*n* = 14), and the average response rate of the 178 students was 10.9% (3.9%).

Over the course of the anatomy curriculum, there were 214 total responses from participatory group members across 53 different students. 63.1% (*n* = 214) of the responses reported that the group facilitator helped the group understand the content better. Thematic analysis of 22 students who answered the survey found that 4 participants reported facilitation improved the discussion either by helping the group stay focused or promoting fluid conversation. Participants also identified some challenges they encountered with the inclusion of the group facilitator role (*n* = 18; Table 2). Notably, a third of the respondents reported that their groups disregarded this role. The most common theme was that the group dynamic was naturally balanced without a leader, but other students noted that the facilitator was unprepared or inexperienced. Future studies could explore this phenomenon and identify what additional factors influence the effectiveness and necessity of a facilitator.

**Table 2:** Major themes identified in free-response questions posed to student participants

Open-ended question about facilitator role (*n* = 22)		*n* (%)
Facilitator factors	Facilitator unprepared	3 (14%)
	Facilitator lacks group communication skills	1 (5%)
Group factors	Group naturally stopped facilitation	7 (32%)
	Facilitator unknown	5 (23%)
	Group personalities prevented facilitation	2 (9%)
Other	Helped keep on task	4 (18%)

There were 57 total facilitator responses across 35 different students. Despite participants reporting that their facilitator increased their understanding of the content, 73.7% of the facilitator responses indicated that this role did not increase their own mastery of the content, and only 12.3% indicated that the facilitator had prepared differently compared to when they were only a participatory group member. Of the 7 students who reported preparing differently, 6 of them reported that they felt the role helped them master the content better.

One limitation of this study was the low response rate. Students may have experienced survey fatigue, which likely contributed to the reduced rates for the latter surveys. A higher response rate might have revealed significant differences, considering the latter four questions had a smaller effect size.

A second limitation was the informal, verbal instruction on the framework for group facilitators, since there was great variety in how facilitators interpreted the responsibilities of this role regarding pre-session preparation and conversation guidance. Thus, future work with this format should include more guidance with leadership techniques in order to provide a more uniform facilitator experience and encourage growth. One option could be to include a training workshop, which could help students identify common issues with student learning and strategies to address them, and this has been shown to improve confidence in leading organized and effective sessions [10].

Furthermore, there should be opportunities to provide feedback throughout the year such that students can improve their leadership skills each time. This could take place through peer evaluations, which is a crucial component of the team-based learning (TBL) modality. This immediate peer feedback could allow students to gain professional competencies and encourage self-reflection [6].

## Supplementary Information

Below is the link to the electronic supplementary material.

Supplementary file1 (DOCX 25.6 KB)

## Data Availability

The datasets generated during and analyzed during the current study are available from the corresponding author on reasonable request.
