# Supplementary material for: Development of Leadership Skills During Anatomy Small-Group Sessions in a Pre-clerkship Medical Curriculum
Source: Med Sci Educ. 2025 Apr 14;35(4):1867–70. doi: 10.1007/s40670-025-02391-y (PMC12532514; doi:10.1007/s40670-025-02391-y)
Supplement: Supplementary file 1 — Supplementary file1 (DOCX 25.6 KB) [file 40670_2025_2391_MOESM1_ESM.docx]

**Supplemental 1:** Facilitator note sheet for Rutgers NJMS medical anatomy curriculum

| **Document Number:** |  | **Team Number:** |  |
| --- | --- | --- | --- |
| **Facilitator Name:** |  | | |
| **Number of Team Members in Attendance:** |  | **How much time did you spend on the team discussion (in minutes)?** |  |
| **Did you go through ALL of the Questions for Application? If not, which ones did you skip and why?** | YES                          NO  Explain: | | |
| **Did you go through the Learning Topics to answer each other’s questions? If not, why?** | YES                          NO  Explain: | | |
| **What topic(s) did your team find the most difficult? Why?** |  | | |
| **Did you feel your team had an active discussion? Why or why not?**  *This includes group members discussing information in the course documents and sharing personal understanding of the material, refer to the documents/resources while making decisions (e.g., answering case or quiz questions), discussing areas group members found difficult and addressing questions group members ask, etc* | YES                          NO  Explain: | | |
| **Please rate how active each team member was in the discussion.**  *Always: Dominating the team discussion, leaving little room for peers to talk.*  *Often: Actively involved in the conversation and allowing peers to talk.*  *Sometimes: Talking periodically and letting the other team lead the conversation*  *Never: No participation.* | Facilitator:      Always           Often           Sometimes           Never  Member 1:     Always           Often           Sometimes           Never  Member 2:     Always           Often           Sometimes           Never  Member 3:     Always           Often           Sometimes           Never  Member 4:     Always           Often           Sometimes           Never  Member 5:     Always           Often           Sometimes           Never  Member 6:     Always           Often           Sometimes           Never  Member 7:     Always           Often           Sometimes           Never | | |
| **What strategies did you use to encourage members to participate more or to make room for others to participate? Select all that apply.**  **Please note that as a facilitator it is expected that you discuss these things with the team members as it will help the facilitator grow as a leader and the member grow as a learner. These are things that will be helpful when entering clerkship and residency.* | - Remind the entire group that students should be participating equally - Deflect a question (e.g., “That is a great question, I’d like to hear from someone who hasn’t contributed to the discussion or anyone else want to answer this question?”) - Call on a quiet student during the session to discuss a topic/question - Discuss (or will discuss) with less active students outside of the team session to encourage more participation - Other (please explain): | | |
